# The Relationship between Adverse Childhood Experiences and Sleep Problems among Adolescent Students: Mediation by Depression or Anxiety

**DOI:** 10.3390/ijerph18010236

**Published:** 2020-12-30

**Authors:** E-Jin Park, Shin-Young Kim, Yeeun Kim, Dajung Sung, Bora Kim, Yerin Hyun, Kyu-In Jung, Seung-Yup Lee, Hayeon Kim, Subin Park, Bung-Nyun Kim, Min-Hyeon Park

**Affiliations:** 1 Department of Psychiatry, Incheon St. Mary’s Hospital, College of Medicine, The Catholic University of Korea, Incheon 400-011, Korea; zahir@catholic.ac.kr; 2 Department of Psychiatry, Eunpyeong St. Mary’s Hospital, College of Medicine, The Catholic University of Korea, Seoul 06591, Korea; helenasykim@gmail.com (S.-Y.K.); catholic1120@gmail.com (Y.K.); jenndj28@gmail.com (D.S.); chiroo3333@naver.com (B.K.); rin9211@naver.com (Y.H.); cki@catholic.ac.kr (K.-I.J.); solero82@gmail.com (S.-Y.L.); hayoning@gmail.com (H.K.); 3 Department of Research Planning, National Center for Mental Health, Seoul 04933, Korea; subin-21@hanmail.net; 4 Department of Psychiatry and Behavioral Science, College of Medicine, Seoul National University, Seoul 08826, Korea; kbn1@snu.ac.kr

**Keywords:** threatening experiences, trauma, insomnia, physical health, mental health, interventions

## Abstract

Adverse childhood experiences (ACEs) are known to be closely related to depression, anxiety and sleep problems. However, it remains unclear whether adolescents with ACEs have sleep problems regardless of depression or anxiety or under a mediating effect from depression or anxiety. Therefore, our aim was to examine whether depression or anxiety mediates the relationship between ACEs and sleep problems in adolescents by using a community sample. The Early Trauma Inventory Self Report–Short Form (ETISR-SF) and List of Threatening Experiences Questionnaire (LTE-Q) were used to assess traumatic ACEs. Ultimately, data from 737 students (M = 448, F = 289, 15.1 ± 1.4 years old) were included in the statistical analysis. A total of 576 (78.1%) participants reported that they had experienced one or more ACEs. Adolescents with ACEs had higher levels of depression, anxiety and sleep problems than did adolescents without ACEs, and boys tended to experience more trauma than girls. Depression and anxiety partially mediated the relationship between ACEs and sleep problems. The results of this study suggest the need for depression and anxiety interventions for adolescents with ACEs to reduce the long-term consequences, including sleep problems and physical health problems.

## 1. Introduction

Adverse childhood experiences (ACEs) are defined as any potentially stressful or traumatic events that occur prior to the age of 18 years [1]. The terms “child abuse” and “ACEs” are often used interchangeably, but the latter includes not only trauma experienced by a child who has been subjected to abuse but also a slightly broader range of trauma that encompasses parental imprisonment, parental mental disorder, parental drug addiction, violence against the mother, family poverty and bullying. The Centers for Disease Control and Prevention (CDC) define ACEs as follows: adverse childhood experiences, or ACEs, are potentially traumatic events that occur in childhood (0–17 years), for example, experiencing violence, abuse, or neglect; witnessing violence in the home or community; and having a family member attempt or die by suicide. Additionally, aspects of the child’s environment that can undermine their sense of safety, stability, and bonding, such as growing up in a household with substance misuse, mental health problems, and instability due to parental separation or household members being in jail or prison, are included [2].

To date, approximately 50% of children in the United States have experienced at least one type of childhood adversity [3,4]. The Korea Institute for Health and Social Affairs reports that approximately 79% of adult parents with children under the age of 18 have been exposed to ACEs, based on the Status Survey on the Life Experience of Children’s Families [5]. However, according to one study [6], approximately 57% of adults had experienced more than one ACE, as based on the Korean General Social Survey.

ACEs are known to have severely harmful effects in terms of mental problems such as depression and anxiety. In fact, adults with a history of ACEs tend to be 1.1–2.7 times more likely to experience lifetime depression than those without a history of ACEs [7]; experiences of ACEs can significantly predict the development of anxiety in adults, and the magnitude of ACEs and anxiety correlate significantly [8].

A large body of epidemiological research has also focused on the relationship between ACEs and sleep problems in adulthood [9,10,11]. Several retrospective studies found that adults with a history of multiple childhood adversities were more likely to have sleep problems than were those without a history of ACEs [9,12,13,14,15]. Moreover, in a comparison of different categories of people with a history of ACEs, those included in more than five categories had 2.1 times more difficulty falling or staying sleep than people without ACE experience [9].

Furthermore, ACEs are known to have a harmful effect not only on mental health but also on physical health, with adverse consequences such as cardiovascular and endocrine disorders [16]. In general, sleep problems are likely to serve as a path that links ACE-related mental and physical health problems.

Sleep problems are symptoms of depressive disorders and anxiety disorders, and the relationship of sleep problems with ACEs, depression and anxiety has been demonstrated in many previous ACE studies. In particular, several recent studies show that sleep problems that occur after experiencing traumatic events such as ACEs increase the risk of trauma-related mental health problems [17]. However, there is insufficient research on the relationship between people with ACEs and sleep problems. Therefore, a detailed evaluation of the relationship between sleep and ACEs, along with the effects of mood symptoms such as depression and anxiety, should be performed. Additionally, ACEs are known to be closely related to depression, anxiety and sleep problems. In addition, adolescent girls tend to have more depression, anxiety and sleep problems, and it is commonly regarded that ACE exposure and the consequences of ACEs are more severe in girls than in boys. However, it remains unclear whether adolescents with ACEs have sleep problems regardless of depression or anxiety or under a mediating effect of depression or anxiety, and if there is a gender difference in ACE exposure and ACE consequences, whether it is a real gender difference, or if this gender difference might result from the mediating effect of depression or anxiety.

Therefore, this study used a community sample to determine (1) whether there are differences in sleep problems between adolescents who have experienced ACEs and those who have not and (2) whether depression or anxiety serves as a mediator of sleep problems in adolescents who have experienced ACEs.

## 2. Materials and Methods

### 2.1. Participants

Participants were recruited from one middle school and one senior high school located in Seoul, South Korea. After the school principals approved the research, the investigators visited the schools, explained the purpose of the study to the students and teachers, and obtained consent. We also mailed letters to the parents to outline the study objectives, guarantee confidentiality, provide a contact telephone number for the principal investigator, and indicate that parents would be informed of the results after the analyses were completed. The letter also included a statement that parents were free to refuse to respond if they did not agree with the study’s objective. The Institutional Review Board (IRB) for Human Subjects at Seoul National University Hospital approved the study protocol. Detailed information about the study was given to the parents and children, and written informed consent was obtained before study entry. Among the students at these schools, a total of 802 students between the 7th and 11th grades (age range: 13–17 years old) volunteered to participate in this study. Parents were asked to complete questionnaires about socio-demographic characteristics (e.g., family income and parental educational level).

### 2.2. Measures

#### 2.2.1. Early Trauma Inventory Self Report–Short Form (ETISR-SF)

The ETISR-SF is a 27-item, self-report questionnaire that assesses the following four domains: physical abuse, emotional abuse, sexual abuse, and general trauma [18]. The ETISR-SF assesses the occurrence of traumatic events in each domain before the age of 18 [19]. Each traumatic experience is scored dichotomously (yes = 1/no = 0), and the sum of the scores of each domain as well as the total scores are calculated.

The ETISR-SF has been validated with Korean adolescent samples, and the internal consistency was high (Cronbach’s *α* = 0.803) [20]. Based on the ETISR-SF responses, the participants who reported experiencing any traumatic events were assigned to the ETI+ group, and those who did not report experiencing any traumatic events were assigned to the ETI− group.

#### 2.2.2. List of Threatening Experiences Questionnaire (LTE-Q)

A modified version of the LTE-Q was used to assess exposure to life-threatening experiences [21]. The LTE-Q is a 12-item instrument used to measure threatening life events experienced over the last six months (the 12 items are as follows: serious illness or injury to the subject; serious illness or injury to a close relative; death of a first-degree relative including a child or spouse; death of close family friend or second-degree relative; separation due to marital difficulties; breaking off a steady relationship; serious problems with a close friend, neighbor or relative; becoming unemployed or seeking work for more than one month; being sacked from one’s job; a major financial crisis; problems with police and court appearance; and something valuable being lost or stolen). The items that were not appropriate for adolescents (e.g., items concerning unemployment or being sacked from one’s job) were modified to be developmentally appropriate. Each life event is scored dichotomously (yes = 1/no = 0); therefore, the total score ranges from 0 to 12. As indicated above, we assigned the participants who reported experiencing any life-threatening events, based on their LTE-Q responses, to an LTE+ group and those who did not report experiencing any life-threatening events to an LTE− group.

#### 2.2.3. Insomnia Severity Index (ISI)

The ISI is a 7-item scale for assessing the perceived severity of insomnia symptoms [22]. The respondents used a 5-point Likert scale ranging from 0 to 4 to describe their insomnia over the past 2 weeks. A previous study indicated high internal consistency for this scale (Cronbach’s *α* = 0.92) [23]. For logistic regression, we assigned the participants into two groups, a sleep problem group and a control group, using a cutoff score of 8 [23].

#### 2.2.4. Epworth Sleepiness Scale (ESS)

Daytime sleepiness was measured using the ESS, a frequently applied scale that rates the likelihood of dozing during 8 daily life situations [24]. Higher scores on the ESS indicate a greater propensity for sleepiness. In the present study, excessive daytime sleepiness was defined as an ESS score of ≥ 8. The Korean version of the ESS has been verified as a reliable and valid measure of daytime sleepiness, and it is commonly used for adolescents (Cronbach’s *α* = 0.73–0.90) [25,26]. When performing logistic regression analyses, we assigned the participants into two groups, a sleep problem group and a control group, using a cutoff score of 11 [24].

#### 2.2.5. School Sleep Habits Survey (SSS)

The SSS assesses usual sleep/wake patterns over the previous two weeks and includes a Sleepiness Scale, Sleep-Wake Behavior Problems Scale, Depressed Mood Scale, and Morningness-Eveningness Scale [27]. During this study, we used only the Sleep-Wake Behavior Problems Scale (SSS_prob).

The SSS_prob contains 15 items that reflect a combination of difficulties with sleep initiation and maintenance as well as other sleep-related problems. Scores range from 10 to 75, with higher scores indicating more sleep problems. The Cronbach’s *α* for the SSS_prob was previously found to be 0.75 [27].

We divided our sample into two groups (i.e., sleep problem and control groups) using a cutoff score of 22 for the SSS_prob for logistic regression analyses. Because it was not possible to obtain official cutoff scores, we used first quartile scores.

#### 2.2.6. Children’s Depression Inventory (CDI)

The CDI is a scale that has been modified from the Beck Depression Inventory to be suitable for children and translated into Korean [28]. The CDI evaluates the level of depression based on self-reports by children and adolescents from 7 to 17 years old; for the CDI scale, the cutoff was 20 points [29].This scale consists of 27 items, and each item is rated from 0 to 2 points according to severity. The total score ranges from 0 to 54 points, and a higher score indicates a more severe level of depression [30]. Participants with high CDI scores were assigned to a depression group (CDI+).

#### 2.2.7. Revised Children’s Manifest Anxiety Scale (RCMAS)

Symptoms of anxiety were assessed using the Korean version of the RCMAS, which was originally developed by Reynolds and Richmond [31,32]. It is a self-report screening tool (Cronbach’s *α* = 0.94) to measure anxiety in children and adolescents aged 6–19. The RCMAS consists of 37 items, each requiring a yes or no answer. Three anxiety subscales are included: physiological anxiety, worry/oversensitivity, and social concerns. A total score of 25 or greater is clinically significant, and children with a total score of 34 or above are referred to a psychiatric clinic for further assessment. In this study, participants were assigned to an anxiety group (RCMAS+) if their total score was 25 or higher.

### 2.3. Statistical Analysis

The hypothesis that anxiety and depression mediate the relationship between ACEs and sleep problems was tested using SPSS PROCESS Macro Model 4 (IBM, Armonk, NY, USA) [33]. To determine the significance of direct and indirect effects, we evaluated 5000 bootstrapped samples, and indirect effects were considered significant when zero was not contained in the 95% confidence intervals. In detail, we employed a regression analysis to test whether adolescents’ sleep problems are predicted by ACEs by linear regression analysis. We also performed linear regression analysis to assess whether depression and/or anxiety are predicted by ACEs. Finally, we examined whether adolescents’ sleep problems are predicted by both ACEs and either depression or anxiety problems by multiple regression analysis.

## 3. Results

Among the initially recruited 802 students (M = 494, F = 308, 15.1 ± 1.4 years old), data for 64 who did not respond to the survey were excluded from the statistical analysis. Therefore, data for 737 students (M = 448, F = 289, 15.1 ± 1.4 years old) were included in the statistical analysis. A total of 576 (78.1%) participants reported that they had experienced one or more traumatic experience(s) (ETI+), and 205 (27.8%) reported that they had experienced threatening life events over the last six months (LTE+). The mean monthly family income of the LTE(+) group was significantly lower than that of the LTE(−) group. There were no differences in parental education level according to LTE or ETI classification. The demographic and basic clinical information for the 737 participants is shown in Table 1.

In general, girls tended to report greater sleep-related problems (ESS, M= 6.3 ± 4.0, F = 7.1 ± 4.3, *p* = 0.006; SSS_prob, M = 17.6 ± 5.5, F = 19.2 ± 5.7, *p* < 0.001; ISI, M = 5.1 ± 4.1, F = 5.7 ± 4.2, *p* = 0.088), depressive symptoms (CDI, M = 14.1 ± 6.9, F = 16.5 ± 7.0, *p* < 0.001) and anxiety symptoms (RCMAS, M = 13.7 ± 6.4, F = 14.9 ± 6.2, *p* = 0.009) than boys.

Conversely, regarding ACEs, the mean ETI total score was significantly higher for boys than for girls (M = 4.1 ± 3.7, F = 3.1 ± 2.9, *p* < 0.001). Furthermore, of the four mean ETI domain scores, the mean scores in the physical abuse, sexual abuse and general trauma domains were significantly higher in boys than in girls (physical abuse: M = 2.0 ± 1.8, F = 1.5 ± 1.6, *p* < 0.001; sexual abuse: M = 0.1 ± 0.4, F = 0.1 ± 0.3, *p* = 0.010; general trauma: M = 1.0 ± 1.5, F = 0.6 ± 0.9, *p* < 0.001), and the percentage of participants who experienced each type of trauma was significantly higher among boys than girls (physical abuse: M = 41.2% vs. F = 22.9%, *p* = 0.010; sexual abuse: M = 6.1% vs. F = 2.2%, *p* = 0.030; general trauma domain: M = 29.7% vs. F = 15.2%, *p* = 0.007). No statistically significant difference between boys and girls was observed for the mean ETI emotional abuse score alone (M = 0.8 ± 1.3, F = 0.9 ± 1.2, *p* = 0.864), with the percentage of participants who experienced emotional abuse not differing significantly between boys and girls (M = 22.9% vs. F = 16.8%, *p* = 0.160). Although there was no difference between boys and girls in the total LTE score (M = 0.4 ± 0.7, F = 0.4 ± 0.7, *p* = 0.554), 5 (1.1%) boys reported experience related to LTE item 10 (problems with police and court appearance), whereas no girls reported experience with this event. To explore the effects of depression or anxiety symptoms on the relationship between ACEs and sleep problems in greater detail, we examined the mediating effects of the CDI or RCMAS scores on the relationship between ACE-related scale scores (ETI and LTE) and sleep-related scale scores (ISI, ESS and SSS_prob).

**Figure 1:**
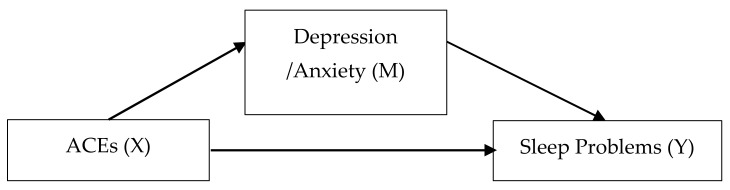
Depressive or anxiety symptoms as mediators between adverse childhood experiences (ACEs) and sleep problems.

Figure 1 shows the hypothesized pathways by which ACEs influence sleep problems, tested by mediation analysis. The upper pathway (ACEs lead to depression/anxiety, which then lead to sleep problems) is the mediated pathway or the indirect effect of ACEs on sleep problems. The lower pathway (ACEs lead directly to sleep problems) is the unmediated pathway, or the direct effect. The total effect of ACEs on sleep problems is estimated using a bivariate linear regression model with the ACE variable as the independent variable and the sleep problem variable as the dependent variable. The direct effect is estimated using a multivariate linear regression model with the ACE variable and the depression/anxiety variable as the independent variables and the sleep problem variable as the dependent variable. The indirect effect is estimated by subtracting the direct effect from the total effect.

We performed the bootstrap procedure [33] to estimate the standard errors and 95% confidence limits of the indirect and direct effects. The bootstrapping we used effectively controls the type I error rate compared to the causal step approach and the Sobel test [34] compared to normality [35], so it is used as a powerful method for analyzing statistical significance. In many studies, the bootstrapping technique is considered a resampling procedure that can take 5000 samples and affect the size estimation that computes the indirect effect for each sample; thus, its confidence interval consists of a 95% confidence interval. As a result, confidence intervals that do not contain zero values are considered statistically significant at *p* < 0.05.

Table 2 shows the results of testing the mediation models, each with one variable representing adverse childhood experience, one variable representing depression or anxiety (the hypothesized mediator), and one variable representing sleep problems. Since there were two possible ACE variables (ETI and LTE), two possible mediator variables (CDI and RCMAS) and three possible sleep problem variables (ISI, ESS, and SSS_prob), we tested 2 × 2 × 3 = 12 models. A model showing a statistically significant indirect (mediated) effect but no statistically significant direct effect would be consistent with full mediation. A model showing a statistically significant indirect (mediated) effect and a statistically significant direct effect would be consistent with partial mediation.

As shown in Table 1 and Table 2, models 1, 2, 3, 4, 5, 7, 9, 10, 11, and 12 showed partial mediation. Model 1’s independent variable ETI significantly predicted the mediator CDI (B = 0.057, *p* < 0.001), and both ETI (B = 2.02, *p* < 0.001) and CDI (B = 0.28, *p* < 0.001) significantly predicted the ISI. The indirect effect was 0.08 and 95% CI (0.05–0.12), and the direct effect was 0.28, 95% CI (0.20–0.37). Since there is no 0 between the 95% LLCI and ULCI of the indirect and direct effects, the partial mediation effect is significant at 0.05. Model 2’s independent variable ETI significantly predicted the mediator RCMAS (B = 0.73, *p* < 0.001), and both ETI (B = 0.16, *p* = 0.003) and RCMAS (B = 0.26, *p* < 0.001) significantly predicted the ISI. The indirect effect was 0.19 and 95% CI (0.15–0.24), and the direct effect was 0.16, 95% CI (0.07–0.24). As there is no 0 between the 95% LLCI and ULCI of the indirect and direct effects, the partial mediation effect is significant at 0.05. Model 3’s independent variable LTE, which significantly predicted the mediator CDI (B = 1.63, *p* < 0.001), and both LTE (B = 1.12, *p* < 0.001) and CDI (B = 0.17, *p* < 0.001) significantly predicted the ISI. The indirect effect was 0.27, 95% CI (0.14–0.42), and the direct effect was 1.13, 95% CI (0.70–1.56). With no 0 between the 95% LLCI and ULCI of the indirect and direct effects, the partial mediation effect is significant at 0.05. Model 4’s independent variable LTE significantly predicted the mediator RCMAS (B = 2.38, *p* < 0.001), and both LTE (B = 0.76, *p* < 0.001) and RCMAS (B = 0.28, *p* < 0.001) significantly predicted the ISI. The indirect effect was 0.66 and 95% CI (0.46–0.88), and the direct effect was 0.77, 95% CI (0.36–1.18). Since there is no 0 between the 95% LLCI and ULCI of the indirect and direct effects, the partial mediation effect is significant at 0.05. Model 5’s independent variable ETI significantly predicted the mediator CDI (B = 0.57, *p* < 0.001), and both ETI (B = 0.16, *p* < 0.001) and CDI (B = 0.08, *p* < 0.001) significantly predicted the ESS. The indirect effect was 0.05 and 95% CI (0.02–0.08), and the direct effect was 0.16, 95% CI (0.07–0.25). Because there is no 0 between the 95% LLCI and ULCI of the indirect and direct effects, the partial mediation effect is significant at 0.05.

Model 7’s independent variable LTE significantly predicted the mediator CDI (B = 1.63, *p* < 0.001), and both LTE (B = 0.70, *p* = 0.002) and CDI (B = 0.09, *p* < 0.001) significantly predicted the ESS. The indirect effect was 0.15 and 95% CI (0.06–0.27), and the direct effect was 0.07, 95% CI (0.26–1.14). Since there is no 0 between the 95% LLCI and ULCI of the indirect and direct effects, the partial mediation effect is significant at 0.05. Model 9’s independent variable ETI significantly predicted the mediator CDI (B = 0.57, *p* < 0.001), and both the ETI (B = 0.36, *p* < 0.001) and CDI (B = 0.11, *p* < 0.001) significantly predicted the SSS_prob. The indirect effect was 0.06 and 95% CI (0.02–0.10), and the direct effect was 0.36, 95% CI (0.24–0.48). Since there is no 0 between the 95% LLCI and ULCI of the indirect and direct effects, the partial mediation effect is significant at 0.05. Model 10’s independent variable ETI significantly predicted the mediator RCMAS (B = 0.74, *p* < 0.001), and both ETI (B = 0.25, *p* < 0.001) and RCMAS (B = 0.21, *p* < 0.001) significantly predicted the SSS_prob. The indirect effect was 0.16 and 95% CI (0.10–0.22), and the direct effect was 0.25, 95% CI (0.12–0.37). Since there is no 0 between the 95% LLCI and ULCI of the indirect and direct effects, the partial mediation effect is significant at 0.05.

Model 11’s independent variable LTE significantly predicted the mediator CDI (B = 1.65, *p* < 0.001), and both the LTE (B = 1.52, *p* < 0.001) and the CDI (B = 0.14, *p* < 0.001) significantly predicted the SSS_prob. The indirect effect was 0.22 and 95% CI (0.10–0.39), and the direct effect was 1.52, 95% CI (0.92–2.12). Since there is no 0 between the 95% LLCI and ULCI of the indirect and direct effects, the partial mediation effect is significant at 0.05. Model 12’s independent variable LTE significantly predicted the mediator RCMAS (B = 2.37, *p* < 0.001), and both LTE (B = 1.13, *p* < 0.001) and RCMAS (B = 0.23, *p* < 0.001) significantly predicted the SSS_prob. The indirect effect was 0.56 and 95% CI (0.35–0.79), and the direct effect was 1.13, 95% CI (0.54–1.72). Since there is no 0 between the 95% LLCI and ULCI of the indirect and direct effects, the partial mediation effect is significant at 0.05.

Finally, Model 6 and 8 showed full mediation. Model 6’s independent variable ETI significantly predicted the mediator RCMAS (B = 0.73, *p* < 0.001). When both ETI and RCMAS were considered, RCMAS (B = 0.17, *p* < 0.001) significantly predicted the ESS, while ETI (B = 0.09, *p* = 0.061) did not significantly predict the ESS. Model 6’s indirect effect was 0.12 and 95% CI (0.08–0.17), while the direct effect was 0.08 and 95% CI (0.0040–0.1764). Since there is 0 only between LLCI and ULCI of the direct effect, this model shows full mediation. Additionally, Model 8’s independent variable LTE significantly predicted the mediator RCMAS (B = 2.38, *p* < 0.001). When both ETI and RCMAS were considered, RCMAS (B = 0.18, *p* < 0.001) significantly predicted the ESS, while LTE (B = 0.27, *p* = 0.230) did not significantly predict ESS. Model 8’s indirect effect was 0.43 and 95% CI (0.29–0.61), while the direct effect was 0.26 and 95% CI (0.18–0.70). Since there is 0 only between LLCI and ULCI of the direct effect, this model shows full mediation.

## 4. Discussion

The results of this study showed that in a community sample adolescents with ACEs were more likely to have depression, anxiety and sleep problems than were adolescents without ACEs.

Can ACEs cause sleep problems regardless of depression or anxiety?

This study revealed dose-response relationships between ACEs and sleep problems, and the result was consistent with a previous study by Chapman et al. reporting a dose-response relationship between ACEs and sleep problems [9]. However, when we controlled for the effect of depressive or anxiety symptoms, the effects of ACEs on sleep problems were smaller. In most (10 of 12) models, we found evidence of partial mediation by depression or anxiety, and in two models we found evidence of full mediation by anxiety. A systematic review of the relationship between ACEs and adult sleep disorders by Kajeepeta et al. indicated that “there are relationships between ACEs and sleep problems regardless of mental health status” [12]. Kajeepeta et al. cited five articles as grounds for their argument [15,36,37,38,39].

Nonetheless, when we examined each cited article in detail, we developed strong doubts about the validity of the argument in Kajeepeta et al. In fact, among the cited articles, the first two, by Chapman et al. [37] and Bader et al. [15] either did not show a relationship between ACEs and sleep problems regardless of mental health status or did not aim to determine whether this relationship exists. Moreover, the studies by Cuddy and Belicki [39] and Noll [36] examined only sexual abuse among the various types of ACEs. Finally, the study by Brower et al. [38] addressed patients with alcohol dependence, and the participants’ mental status was measured using only the Short Form-36 Physical and Mental Health Summary Scales. Overall, we determined that the five cited studies could not provide sufficient support for the argument that “there are relationships between ACEs and sleep problems regardless of mental health status” with regard to general ACEs, except for sexual abuse; thus, it is difficult to state that this argument is definitely valid. Additionally, regarding sexual abuse among ACEs, the result that “sexual abuse can cause sleep problems regardless of mental health status” has been replicated several times [36,39].

Our results that depression or anxiety mediates the relationship between ACEs and sleep problems are supported by objective biological studies using polysomnography or actigraphy. Compared with adults without ACEs, adults with ACEs tend to have higher sleep onset latency, number of awakenings, number of movement arousals, number of body movements, proportion of sleep time spent moving, and proportion of sleep time spent in rapid eye movement (REM) sleep as well as lower sleep efficiency [15,40,41,42]. These findings are consistent with those commonly observed among people with depression or anxiety [42]. It is important not to over-generalize. Differences between our findings and the other cited studies were likely due to differences in methods, including the populations studied, and the choice of measurement variables used to represent adverse childhood experiences, mental health, and sleep problems.

Are there any sex differences in the relationship between ACE and sleep problems?

In general, women are reported to experience more trauma than men, and Frazer et al. argued that women have as many as three times more traumatic experiences. Regardless, prior studies have reported many male adult, adolescent, and child traumas [43,44].

It is generally accepted that adolescent girls tend to have more depression, anxiety and sleep problems than adolescent boys, and the results of this study were consistent with those findings. Nevertheless, our study showed that the consequences of ACEs in boys are not as minor as those in girls. Furthermore, the exposure rate to ACEs might be higher in boys than in girls. The high prevalence rate of ACE in this study is mainly due to the broad nature of the ETI scale, which, in this study, counted even a minor trauma in childhood as an ACE. The prevalence rate of 78.1% measured in this study is similar to that of youth or young adults in community samples from Asian countries, which ranged from 76% to 79% using a broad measurement of ACEs [45,46], although the observation that the ETI scores were higher for boys than for girls is remarkable. In particular, ACEs of a physical rather than emotional nature were dramatically higher in boys than in girls. In other words, the prevalence of ACEs by sex might vary according to the definition of ACEs. However, a stereotype that ACE exposure and the consequences of ACEs are higher in girls than in boys might exist [47], and there is also a stereotype that women are much more vulnerable to the development of depression, anxiety or sleep problems after experiencing an ACE than are men [7].

We cannot overlook the importance of studies that have reported that rates of ACE experiences are higher in men than in women [43,44]. In particular, sexual abuse in boys has been seriously underreported [48].

What is the relationship between ACEs and physical health?

There is considerable evidence that there are substantial similarities between the mechanisms through which ACEs cause sleep problems and those by which depression or anxiety cause sleep problems. As mentioned above, polysomnography and actigraphy findings are very similar between adults with depression or anxiety and adults with ACEs.

Regarding the biological mechanism by which ACEs cause sleep problems, “altered corticotropin-releasing hormone (CRH) reactivity related to altered hypothalamic-pituitary-adrenal (HPA) axis functioning induced by ACEs may contribute to alterations in sleep regulation”, which is a well-recognized biological mechanism that is commonly observed in people with depression or anxiety [12]. According to previous electroencephalography (EEG) studies [12,49], another possible mechanism by which ACEs cause sleeping problems is neural hyperactivity, which is also commonly observed in people with anxiety.

Many studies have argued that there is a close relationship between ACEs and poor physical health: premature mortality [16,50,51], metabolic syndrome [52,53], obesity [54] and cardiovascular disease [55] are commonly mentioned ACE-related health problems. In addition, a low blood concentration of natural killer cells can reportedly cause immune dysfunction in people with ACEs [56].

As one of the etiologies of ACEs related to poor physical health, adverse lifestyles, such as stress-induced eating [52,57], physical inactivity [58], alcohol abuse [59] smoking [46], and illicit drug use [60], are commonly observed in those with ACEs compared to those without ACEs. Moreover, people with ACEs tend to have lower socioeconomic status (SES) than people without ACEs [61], and lower SES is closely related to poor physical health [62].

When assessing ACEs, related risk factors that cause poor physical health are widely regarded as risk factors related to depression and anxiety. Therefore, we can infer that not only sleep problems but also, more robustly, depression and anxiety serve as mediators between ACEs and poor physical health.

Our findings must be considered in the light of several limitations. First, due to the nature of the cross-sectional study design, we were not able to address the causality between ACEs and sleep problems. Second, recall bias might have affected the association between ACEs and current sleep problems. Third, our findings might be subject to sampling bias because our sample was obtained in two regions in Seoul. Fourth, in this study, a self-report scale was used. Self-report scales should take into account that response biases may appear. Nevertheless, the sample may reflect the natural distribution of depression and anxiety prevalence in community settings [17].

## 5. Conclusions

We found a high prevalence of sleep problems among adolescents with ACEs, and mediating effects of depression and anxiety were detected in the relationship between ACEs and sleep problems in the adolescents in this community sample. To prevent lifelong effects of ACEs on public health, it is essential to identify and address possible mechanisms linking ACEs and sleep problems. The results of this study suggest that boys’ mental health and sleep problems are greatly affected by ACEs, and it is difficult to say whether boys are more resilient to ACE-related consequences than girls. In addition, depression and anxiety play very important roles in ACE-related sleep problems. The findings suggest that interventions to prevent the development of depression and anxiety following ACEs might be helpful not only for mental health but also for physical health.

## Figures and Tables

**Table 1:** Demographic and clinical characteristics by history of adverse childhood experiences.

Variables		ETI+ (*N* = 576)	ETI− (*N* = 161)	t, F, χ^2^	*p*	*p* ^†^
		M (SD), *N*	M (SD), *N*			
Age		15.06 (1.39)	15.14(1.30)	−0.673	0.501	—
Sex	Boys	356	92	1.148	0.284	0.284
	Girls	220	69			
Monthly family income ^1^		270.00 (155.46)	313.03 (182.20)	−1.099	0.274	
Parental education (years)	Mother	12.44 (2.45)	12.67 (2.50)	0.656	0.513	
	Father	12.83 (2.59)	13.15 (2.69)	0.765	0.445	
ISI		5.64(4.23)	4.22 (3.82)	3.854	<0.001 ***	<0.001 ***
ESS		6.90 (4.24)	5.60 (3.58)	3.883	<0.001 ***	<0.001 ***
SSS_prob		18.67 (5.78)	16.61(4.77)	4.622	<0.001 ***	<0.001 ***
CDI		15.51 (7.04)	13.43 (6.70)	3.230	0.001 **	0.001 **
RCMAS		14.88 (6.29)	11.63 (6.05)	5.832	<0.001 ***	<0.001 ***
**Variables**		**LTE+** **(*N* = 205)**	**LTE−** **(*N* = 532)**	**t, F, χ^2^**	***p***	***p*^†^**
		**M (SD), *N***	**M (SD), *N***			
Age		15.04 (1.38)	15.10 (1.36)	−0.520	0.603	—
Sex	Boys	120	328	0.559	0.437	0.437
	Girls	85	204			
Monthly family income ^1^		252.86 (182.28)	322.48 (174.20)	2.034	0.044	
Parental education (years)	Mother	12.51 (2.50)	12.48 (2.44)	−0.115	0.908	
	Father	12.90 (2.59)	12.88 (2.62)	−0.060	0.952	
ISI		6.74 (4.77)	4.79 (3.79)	5.233	<0.001 ***	<0.001 ***
ESS		7.40 (4.00)	6.30 (4.15)	3.247	0.001 **	0.001 **
SSS_prob		20.11 (5.90)	17.50 (5.36)	5.772	<0.001 ***	<0.001 ***
CDI		16.90 (7.16)	14.34 (6.84)	4.345	<0.001 ***	<0.001 ***
RCMAS		16.56 (6.15)	13.25 (6.22)	3.230	<0.001 ***	<0.001 ***

Note. ETI+, ETI−: Based on the ETISR-SF results, we assigned the participants who reported experiencing any traumatic events to the ETI+ group and those who did not report experiencing any traumatic events to the ETI− group. LTE+, LTE−: Based on the LTE-Q results, we assigned the participants who reported experiencing any life-threatening events to the LTE+ group and those who did not report experiencing any life-threatening events to the LTE− group. ISI = Insomnia Severity Index; ESS = Epworth Sleepiness Scale; SSS_prob = School Sleep Habits Survey, Sleep-Wake Problems Behavior Scale; CDI = Children’s Depression Inventory; and RCMAS = Revised Children’s Manifest Anxiety Scale. ^1^ Unit = 1000 won, which is equivalent to approximately. 1 U.S. dollar. *p* ^†^ = age adjusted. ** *p* < 0.01, *** *p* < 0.001.

**Table 2:** Mediating effects of anxiety and depression between ACEs and sleep problems.

Model	Variables	Mediator Variable: CDI			Dependent Variable: ISI		
**1**		B	S.E	t	B	S.E	t
	Constant	12.94	0.38	34.45	2.02	0.35	5.80
	ETI	0.57	0.07	7.76	0.28	0.04	6.56
	CDI				0.15	0.02	6.77
		$R^{2}$ = 0.08, *F* = 60.16, *p* < 0.001			$R^{2}$ = 0.15, *F* = 62.15, *p* < 0.001		
		**Effect**	**Boot SE**		**95%** **Boot LLCI**		**95%** **Boot ULCI**
	Indirect effect	0.0831	0.0169		0.0536		0.1207
	Direct effect	0.2827	0.0431		0.1981		0.3673
**2**		**Mediator Variable: RCMAS**			**Dependent Variable: ISI**		
		B	S.E	t	B	S.E	t
	Constant	11.48	0.32	36.41	1.01	0.33	3.04
	ETI	0.73	0.06	11.72	0.16	0.04	3.61
	RCMAS				0.26	0.02	11.34
		$R^{2}$ = 0.16, *F* = 137.43, *p* < 0.001			$R^{2}$ = 0.22, *F* = 103.42, *p* < 0.001		
		**Effect**	**Boot SE**		**95%** **Boot LLCI**		**95%** **Boot ULCI**
	Indirect effect	0.1936	0.0240		0.1496		0.2439
	Direct effect	0.1552	0.0430		0.0709		0.2396
**3**		**Mediator Variable: CDI**			**Dependent Variable: ISI**		
		B	S.E	t	B	S.E	t
	Constant	14.45	0.30	47.82	2.32	0.35	6.71
	LTE	1.63	0.39	4.16	1.12	0.22	5.15
	CDI				0.17	0.02	7.95
		$R^{2}$ = 0.02, *F* = 17.27, *p* < 0.001			$R^{2}$ = 0.13, *F* = 52.59, *p* < 0.001		
		**Effect**	**Boot SE**		**95%** **Boot LLCI**		**95%** **Boot ULCI**
	Indirect effect	0.2728	0.0725		0.1434		0.4229
	Direct effect	1.1281	0.2184		0.6993		1.5570
**4**		**Mediator Variable: RCMAS**			**Dependent Variable: ISI**		
		B	S.E	t	B	S.E	t
	Constant	13.28	0.26	51.19	1.12	0.33	3.37
	LTE	2.38	0.34	7.10	0.76	0.21	3.68
	RCMAS				0.28	0.02	12.55
		$R^{2}$ = 0.06, *F* = 50.34, *p* < 0.001			$R^{2}$ = 0.22, *F* = 103.87, *p* < 0.001		
		**Effect**	**Boot SE**		**95%** **Boot LLCI**		**95%** **Boot ULCI**
	Indirect effect	0.6604	0.1071		0.4602		0.8824
	Direct effect	0.7686	0.2078		0.3606		1.1766
**5**		**Mediator Variable: CDI**			**Dependent Variable: ESS**		
		B	S.E	t	B	S.E	t
	Constant	12.94	0.38	34.49	4.78	0.36	13.14
	ETI	0.57	0.07	7.76	0.16	0.04	3.51
	CDI				0.08	0.02	3.57
		$R^{2}$ = 0.08, *F* = 60.26, *p* < 0.001			$R^{2}$ = 0.05, *F* = 17.53, *p* < 0.001		
		**Effect**	**Boot SE**		**95%** **Boot LLCI**		**95%** **Boot ULCI**
	Indirect effect	0.0457	0.0150		0.0186		0.0776
	Direct effect	0.1576	0.0449		0.0694		0.2458
**6**		**Mediator Variable: RCMAS**			**Dependent Variable: ESS**		
		B	S.E	t	B	S.E	t
	Constant	11.48	0.31	36.49	3.86	0.36	10.84
	ETI	0.73	0.06	11.74	0.09	0.05	1.88
	RCMAS				0.17	0.02	6.89
		$R^{2}$ = 0.15, *F* = 137.79, *p* < 0.001			$R^{2}$ = 0.09, *F* = 36.37, *p* < 0.001		
		**Effect**	**Boot SE**		**95%** **Boot LLCI**		**95%** **Boot ULCI**
	Indirect effect	0.1258	0.0224		0.0845		0.1727
	Direct effect	0.0862	0.0459		−0.0040		0.1764
**7**		**Mediator Variable: CDI**			**Dependent Variable: ESS**		
		B	S.E	t	B	S.E	t
	Constant	14.45	0.30	47.90	4.91	0.36	13.71
	LTE	1.63	0.39	4.16	0.70	0.23	3.13
	CDI				0.09	0.02	4.26
		$R^{2}$ = 0.02, *F* = 17.31, *p* < 0.001			$R^{2}$ = 0.05, *F* = 16.44, *p* < 0.001		
		**Effect**	**Boot SE**		**95%** **Boot LLCI**		**95%** **Boot ULCI**
	Indirect effect	0.1486	0.0531		0.0591		0.2686
	Direct effect	0.0717	0.2255		0.2589		1.1446
**8**		**Mediator Variable: RCMAS**			**Dependent Variable: ESS**		
		B	S.E	t	B	S.E	t
	Constant	13.28	0.26	51.33	3.92	0.36	11.00
	LTE	2.38	0.34	7.10	0.27	0.22	1.20
	RCMAS				0.18	0.02	7.73
		$R^{2}$ = 0.06, *F* = 50.39, *p* < 0.001			$R^{2}$ = 0.08, *F* = 35.17, *p* < 0.001		
		**Effect**	**Boot SE**		**95%** **Boot LLCI**		**95%** **Boot ULCI**
	Indirect effect	0.4364	0.0826		0.2876		0.6130
	Direct effect	0.2614	0.2226		−.1756		0.6984
**9**		**Mediator Variable: CDI**			**Dependent Variable: SSS_prob**		
		B	S.E	t	B	S.E	t
	Constant	12.93	0.37	34.54	15.12	0.49	30.68
	ETI	0.57	0.07	7.73	0.36	0.06	5.96
	CDI				0.11	0.03	3.60
		$R^{2}$ = 0.08, *F* = 59.72, *p* < 0.001			$R^{2}$ = 0.09, *F* = 32.98, *p* < 0.001		
		**Effect**	**Boot SE**		**95%** **Boot LLCI**		**95%** **Boot ULCI**
	Indirect effect	0.0621	0.0198		0.0247		0.1031
	Direct effect	0.3629	0.0609		0.2434		0.4824
**10**		**Mediator Variable: RCMAS**			**Dependent Variable: SSS_prob**		
		B	S.E	t	B	S.E	t
	Constant	11.48	0.31	36.55	14.32	0.48	29.78
	ETI	0.74	0.06	11.79	0.25	0.06	3.96
	RCMAS				0.21	0.03	6.28
		$R^{2}$ = 0.16, *F* = 139.05, *p* < 0.001			$R^{2}$ = 0.11, *F* = 44.60, *p* < 0.001		
		**Effect**	**Boot SE**		**95%** **Boot LLCI**		**95%** **Boot ULCI**
	Indirect effect	0.1554	0.0298		0.1000		0.2153
	Direct effect	0.2454	0.0620		0.1237		0.3671
**11**		**Mediator Variable: CDI**			**Dependent Variable: SSS_prob**		
		B	S.E	t	B	S.E	t
	Constant	14.42	0.30	47.88	15.51	0.49	31.77
	LTE	1.65	0.39	4.21	1.52	0.31	4.95
	CDI				0.14	0.03	4.59
		$R^{2}$ = 0.03, *F* = 17.76, *p* < 0.001			$R^{2}$ = 0.07, *F* = 27.07, *p* < 0.001		
		**Effect**	**Boot SE**		**95%** **Boot LLCI**		**95%** **Boot ULCI**
	Indirect effect	0.2242	0.0736		0.0976		0.3873
	Direct effect	1.5213	0.3073		0.9179		2.1247
**12**		**Mediator Variable: RCMAS**			**Dependent Variable: SSS_prob**		
		B	S.E	t	B	S.E	t
	Constant	13.30	0.26	51.34	14.48	0.48	30.19
	LTE	2.37	0.34	7.08	1.13	0.30	3.78
	RCMAS				0.23	0.03	7.34
		$R^{2}$ = 0.06, *F* = 50.06, *p* < 0.001			$R^{2}$ = 0.11, *F* = 43.90, *p* < 0.001		
		**Effect**	**Boot SE**		**95%** **Boot LLCI**		**95%** **Boot ULCI**
	Indirect effect	0.5554	0.1113		0.3540		0.7881
	Direct effect	1.1323	0.2996		0.5441		1.7204

Note. LLCI = lower limit of confidence interval, ULCI = upper limit of confidence interval, ETI = Early Trauma Inventory, RCMAS = Revised Children’s Manifest Anxiety Scale, ESS = Epworth Sleepiness Scale, ISI = Insomnia Severity Index, SSS_prob, SSS_Prob = School Sleep Habits Survey Sleep—Wake Problems Behavior Scale, LTE = List of Threatening Experiences, and CDI = Children’s Depression Inventory.

## Data Availability

The data that support the findings of this study are available from the corresponding author, upon reasonable request.
